# Effects of mining activities on fish communities and food web dynamics in a lowland river

**DOI:** 10.1002/ece3.11111

**Published:** 2024-03-11

**Authors:** Kristin Scharnweber, Carolin Scholz, Victor Schippenbeil, Stefania Milano, Daniel Hühn

**Affiliations:** ^1^ Plant Ecology and Nature Conservation University of Potsdam Potsdam Germany; ^2^ Ecological Research Station Rees University of Cologne Rees‐Bienen Germany; ^3^ Leibniz Institute for Zoo and Wildlife Research, Evolutionary Ecology Berlin Germany; ^4^ Faculty of Mathematics and Science II, Geography Department Humboldt‐Universität zu Berlin Berlin Germany; ^5^ Potsdam Institute of Inland Fisheries Potsdam Germany

**Keywords:** acid mining drainage, aquatic‐terrestrial coupling, benthic invertebrates, browning, metabarcoding, δ^2^H

## Abstract

Fish communities of streams and rivers might be substantially subsidized by terrestrial insects that fall into the water. Although such animal‐mediated fluxes are increasingly recognized, little is known about how anthropogenic perturbations may influence the strength of such exchanges. Intense land use, such as lignite mining, may impact a river ecosystem due to the flocculation of iron (III) oxides, thus altering food web dynamics. We compared sections of the Spree River in North‐East Germany that were greatly influenced by iron oxides with sections located downstream of a dam where passive remediation technologies are applied. Compared to locations downstream of the dam, the abundance of benthic macroinvertebrates at locations of high iron concentrations upstream of the dam was significantly reduced. Similarly, catch per unit effort of all fish was significantly higher in locations downstream of the dam compared to locations upstream of the dam, and the condition of juvenile and adult piscivorous pike *Esox lucius* was significantly lower in sections of high iron concentrations. Using an estimate of short‐term (i.e., metabarcoding of the gut content) as well as longer‐term (i.e., hydrogen stable isotopes) resource use, we could demonstrate that the three most abundant fish species, perch *Perca fluviatilis*, roach *Rutilus rutilus*, and bleak *Alburnus alburnus*, received higher contributions of terrestrial insects to their diet at locations of high iron concentration. In summary, lotic food webs upstream and downstream of the dam greatly differed in the overall structure with respect to the energy available for the highest tropic levels and the contribution of terrestrial insects to the diet of omnivorous fish. Therefore, human‐induced environmental perturbations, such as river damming and mining activities, represent strong pressures that can alter the flow of energy between aquatic and terrestrial systems, indicating a broad impact on the landscape level.

## Graphical abstract

We demonstrate the ability of mining activities to restructure whole aquatic food webs, altering the fluxes of energy between adjacent systems.

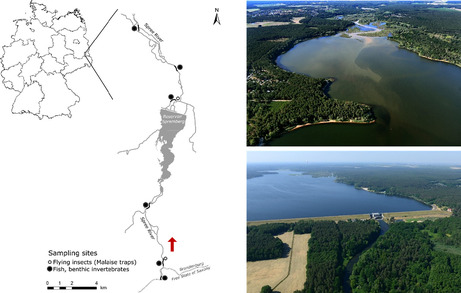

## 1 INTRODUCTION

In the current era of the Anthropocene, river ecosystems worldwide are extensively modified by human activities (Cid et al., 2022; Dudgeon et al., 2006). Land‐use practices are one of the main threads that lead to the loss of freshwater biodiversity (Dudgeon, 2019). Besides the direct negative effect on the overall abundance and richness of organisms, indirect effects may co‐occur by altering the linkages among habitats and communities (Foley et al., 2005). Rivers and their adjacent terrestrial zones are tightly linked via multiple fluxes and pathways (Bartels et al., 2012; Baxter et al., 2005; Polis & Hurd, 1996). In this study, we focus on the animal‐mediated flux from land to water, which is the one of terrestrial invertebrates falling into water bodies and being eaten by fish. This flux can be pronounced and may provide up to 50% of the annual energy budget of fish individuals (Baxter et al., 2005; Nakano & Murakami, 2001). While it has been shown that even the nutrient pool of a lake can substantially be subsidized by terrestrial phosphorus excreted by fish feeding on surface insects (Mehner et al., 2005), these fluxes are generally assumed to be strongest in aquatic systems with extended riparian zones, especially in streams with a high canopy cover (Edwards & Huryn, 1996; Kawaguchi & Nakano, 2001). Although this animal‐mediated flux between adjacent ecosystems is increasingly recognized, little is known about how anthropogenic perturbations may influence the strength of such exchanges (but see Larsen et al., 2016). For example, the degradation of riparian zones, such as from livestock grazing (Saunders & Fausch, 2012) or deforestation (Kawaguchi & Nakano, 2001), significantly decreased the reliance of fish on terrestrial insects. However, another aspect of land use with a strong impact on our river ecosystems and the potential to alter food web dynamics is the one from mining activities.

**FIGURE 1:**
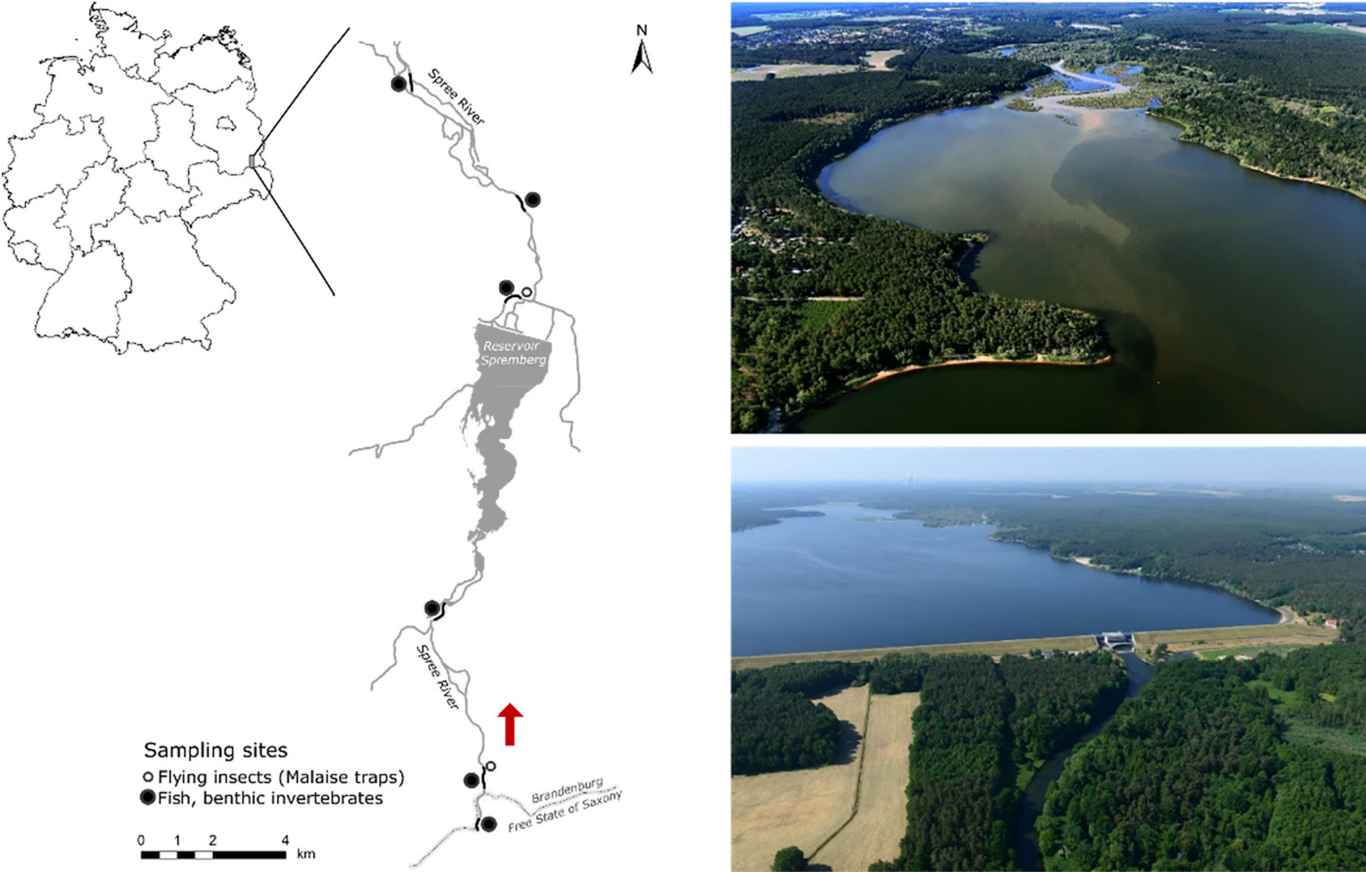
Map of study area, including the location of sampling sites. Photos showing the Spremberg Reservoir with inflow of brown, iron‐rich water (upper photo), and the dam with outflow of clear water (lower photo). Red arrow depicts flow direction. Photos were taken in 2020 and 2021 and kindly supplied by the Lausitzer und Mitteldeutsche Bergbauverwaltungsgesellschaft mbH, Senftenberg/Germany.

Lignite (brown coal) mining activities on aquatic systems are influencing the chemical, physical, biological, and ecological properties of the system (Byrne et al., 2012; Gray, 1997), and iron is one of the key contaminants involved. Due to the oxidation of ferrous sulfide metals (e.g., pyrite and marcasite) during mining or related to the progressive groundwater resurgence in mine reclamation areas, large amounts of iron contaminate groundwater and adjacent aquatic systems. The well‐oxygenated and neutral pH conditions in lotic systems will evoke a flocculation of iron (III) oxides, leading to browning and a turbid environment (Byrne et al., 2012; Gray, 1997; Figure 1). Besides potential toxic effects via direct metal uptake (Vuori, 1995), river organisms suffer from the formation of precipitates covering gills and eggs (Andersson & Nyberg, 1984; Gerhardt, 1992). However, most dominantly, iron precipitates alter the habitat availability for stream organisms as the fine iron sediments bury hard substrates, periphyton, organisms, and clog interstitial spaces of the benthic habitat (Letterman & Mitsch, 1978; McKnight & Feder, 1984). The communities of benthic invertebrates in river sections characterized by high iron oxide concentrations are therefore typically found to be of lower abundance, species biomass, and diversity (Cadmus et al., 2016; Maret et al., 2003; Rasmussen & Lindegaard, 1988; Vuori, 1995). In turn, this could have consequences for the food availability of fish inhabiting the river sections of high iron concentrations, potentially limiting the trophic transfer and the energy available for fish production. Furthermore, we suggest that iron has the potential to restructure the lotic food webs and rearrange the connections to the terrestrial systems. It can be expected that fish inhabiting river sections where benthic invertebrates, as food is scarce, rely to a higher degree on terrestrial invertebrate food resources.

A straightforward approach to estimating the contribution of terrestrial insects to the diet of fish is to simply look at the gut content (Manko, 2016). However, the identification of different prey items can be problematic in cyprinid fishes that have pharyngeal teeth that crush the food. Here, dietary studies using DNA metabarcoding can provide a high resolution of resource use (Amundsen & Sánchez‐Hernández, 2019). Nonetheless, DNA metabarcoding of gut content analyses only allows a snapshot of the recently ingested prey. In addition, stable isotopes can provide an integrated estimate of resource use over longer time periods (Boecklen et al., 2011; Grey, 2006). In the context of identifying the contribution of terrestrial insects to the diet of fish, stable isotopes of hydrogen (δ^2^H) are particularly useful due to the strong divergence between aquatic and terrestrial endmembers (Doucett et al., 2007; Vander Zanden et al., 2016).

Here, we studied the cumulative effects of iron (III) oxides on a lotic food web and the linkages to the terrestrial surroundings. Specifically, we predict that in river sections of high iron concentrations (i.e. upstream of the dam), fewer benthic invertebrates are present, and fish feed on terrestrial insects to a higher degree compared to fish individuals found in river sections of lower iron concentrations (i.e. downstream of the dam, following water remediation). This will be indicated by a higher number of terrestrial species found in the gut content (identified by their DNA sequences using metabarcoding techniques), and further, by a higher terrestrial index calculated from hydrogen stable isotope values demonstrating the longer‐term resource use. In addition, we predict that a low abundance of benthic invertebrates upstream of the dam will decrease the general fish abundance and hence the condition factor of the top predator (piscivorous pike *Esox lucius*).

## 2 METHODS

### 2.1 Study area

We conducted a study at the Spree River, which is a sixth‐order lowland river located in north‐eastern Germany. It flows through the Lusatian region, where lignite mining activities during the times of the German Democratic Republic have been extensive and are still conducted to date (Krümmelbein et al., 2012). In the 1960s, the Spree River was dammed for flood control purposes and hydropower generation. As a consequence, the Spremberg Reservoir was built, which is one of the largest reservoirs in Germany. Nowadays, the reservoir plays a crucial role in securing the water quality of the Spree River impacted by mining activities. Due to the post‐mining groundwater rise, sections of the rivers are characterized by large amounts of iron, sulfate, trace metals, and aluminium (Friedland et al., 2021). To retain the iron load and safeguard the drinking water supply, passive remediation technologies are applied. Sequential conditioning of the river water comprised the addition of lime to increase pH to accelerate iron (II) oxidation and the addition of a flocculation aid to enhance flocculation of iron hydroxide sludge (Uhlig et al., 2016). The precipitated iron sludge usually ranges between 1000 and 4000 kg, occasionally 150,000 kg on a daily basis (Uhlmann et al., 2021). The visibility threshold of the dominant fraction of iron in the study area, iron (III) hydroxide, is about 2–3 mg L^−1^ (Benthaus & Totsche, 2015); hence, the conditioning of the iron‐rich water leads to contrasting environments in river sections with turbid water of high iron concentrations upstream of the dam and clear water of lower iron concentrations downstream of the dam (Friedland et al., 2021; Uhlmann et al., 2021; Figure 1).

### 2.2 Field sampling

All sampling was conducted in summer, between August 16 and 27 2021. Sampling sites were located along a stretch of the Spree River of about 20 km, close to the town of Spremberg, Brandenburg, Germany, where the dam is located (Figure 1). We sampled at six locations in total, three located upstream of the dam (i.e., at total iron (TFe) >3.8 mg L^−1^; Zerre, Trattendorf, Wilhelmstal), and three downstream of the dam (i.e., at TFe < 0.3 mg L^−1^; Bräsinchen, Frauendorf, Madlow). Besides the water quality, sites were comparable with regard to habitat characteristics (Table 1). For three sites (Zerre, Wilhelmstal and Bräsinchen), data regarding turbidity, TFe, total phosphorus, and total nitrogen (TN) were obtained from the local authority (Landesamt für Umwelt Brandenburg) which collects this data monthly within the context of the European water framework directive. We collected benthic macroinvertebrates for abundance estimates and stable isotope analysis from river sediments using an Ekman grab. Samples were sieved through 500 μm and stored in ethanol. In the lab at the Potsdam Institute of Inland Fisheries, they were sorted into broad taxon groups, and individuals were counted. Abundances were expressed as individuals per m^−2^ and for every location an average of three samples was calculated for each taxon group. Seston samples for stable isotopes were collected using a 100 μm plankton net that was lowered into the water. Samples included few and small zooplankton. We used Malaise traps without any collecting agent to sample flying terrestrial insects for the stable isotope analysis (i.e., Tineidea, Diptera, and Tipulidae), which we employed for 24 h. Permit for sampling was received from the respective authority (Landesamt für Umwelt Brandenburg). In addition, single groups (i.e., Formicidae and Orthoptera) were hand‐collected from the meadows. Terrestrial insects were collected at two locations only: at Trattendorf, representing insects upstream of the dam, and at Bräsinchen, representing insects downstream of the dam (Figure 1). Insects were killed by freezing, sorted into groups, and stored at −20°C prior to isotope analyses.

**TABLE 1:** Description of the field sites. Values for total iron (TFe), total nitrogen (TN), total phosphorus (TP), and turbidity represent averages of monthly water samples between April and August 2021. FNU: Formazine Turbidity Unit. Depth near shore indicates the steepness of the shoreline.

Location	Site	Location	TFe (mg L ^−1^)	TN (mg L ^−1^)	TP (μg L ^−1^)	Wetted with (m)	Turbidity (FNU)	Depth near shore (m)	Flow velocity (m s^−1^)	Primary substrate	Aquatic vegetation
Upstream of the dam	Zerre	Above dam	4.1	1.6	29.2	22	22.2	1.5	0.2–0.5	Gravel	Infrequent
	Wilhelmstal	Above dam	3.8	2.1	38.2	22	37.8	1.1	0.2–0.5	Gravel	Infrequent
	Trattendorf	Above dam	–	–	–	24	–	1.4	0.2–0.5	Sand	Frequent
Downstream of the dam	Bräsinchen	Below dam	0.3	1.7	17.2	28	3.1	1.7	0.2–0.5	Sand	Frequent
	Frauendorf	Below dam	–	–	–	28	–	1.2	0.2–0.5	Gravel	Infrequent
	Madlow	Below dam	–	–	–	29	–	1.2	0.2–0.5	Gravel	Infrequent

Electrofishing to sample the fish communities was conducted under permit number 2‐2021 approved by the local environmental agency (hunting and fisheries authority of the administrative district Spree‐Neiße). Sampling was performed at both river banks during the day‐time using a generator‐powered DC electrofishing unit (Type FEG 8000, 8 kW, EFKO Elektrofischfanggeräte GmbH, Leutkirch im Allgäu, Germany) equipped with one anodic hand net of 45 cm ring diameter and a 4 m long copper litz wire as the cathode. Length of electro‐fished river sections ranged between 300 and 640 m at the six locations. Each section was fished in both flow directions (i.e., upstream and downstream) in a standardized way according to the European standard protocol EN 14011:2000 and this procedure was repeated 3 times. Natural boundaries of each section (i.e. weirs or riffles) prevented fish from escaping from electro‐fishing. At each section, captured fishes were identified to species level, counted, measured (up to 1 mm), weighted (up to 0.1 g), and placed in a holding net. Once completed, all captured fishes were released, unless they were taken for further analyses. We summed all fishes caught at one particular section and calculated catch per unit effort (CPUE, expressed as individuals 1000 m^−1^) and biomass per unit effort (BPUE, g 1000 m^−1^). Individuals of the most abundant fish species caught at Trattendorf (upstream of the dam) and Bräsinchen (downstream of the dam) were stored on ice and brought to the lab. Here, gut content was collected for metabarcoding and a small piece of dorsal muscle tissue was sampled for stable isotope analyses of hydrogen. Scales of pike were collected to allow age estimates via counting of annuli in this top predator. To compare the condition of the top predator, and thus, allow a comparison for the amount of energy that reaches the highest trophic levels, we calculated Fulton's condition factor (*K*) according to the Htun‐Han (1978) equation for pike of the different age cohorts:

$$K=\frac{W\times 100\;}{L^{3}},$$

where *W* = weight of fish (g) and *L* = total length of fish (g).

### 2.3 Metabarcoding

We followed the methodological approach described in Scholz and Voigt (2022). In brief, DNA was extracted from the frozen fish gut content by applying NucleoSpin© Food and NucleoSpin© Soil Kit (Macherey‐Nagel GmbH & KG, Düren, Germany) as outlined in the manufacturer's instructions. We performed two DNA extractions for each gut content sample. The concentration of the extracts was determined by fluorometric quantification in a Qubit Fluorometer (Qubit fluorometric quantification dsDNA High Sensitivity Kit, Thermo Fisher Scientific, Walham, USA). Some of the DNAs had to be cleaned and concentrated using a DNA Clean and Concentrator Kit (Zymo Research, 17062 Murphy Ave, Irvine, CA 92614, USA) to get rid of PCR‐inhibitors. Throughout the laboratory work, we strictly applied protocols to prevent contaminations by alien DNA or PCR products. The presence of contaminations was checked through all laboratory steps using different negative controls.

We performed a double‐PCR strategy with dual indexing. The first PCR amplified the target region CO1 (Cytochrome oxidase subunit 1) region (Galan et al., 2018), the second PCR added the indices to the target region. Products were checked with an agarose gel and cleaned twice with magnetic beads (CleanNGS, GC biotech, Waddinxveen, Niederlande). All products were quantified by fluorometric quantification in the plate reader (Quant‐iT™ dsDNA Assay Kit, high sensitivity, Thermo Fisher Scientific, Walham, USA) and pooled in equimolar concentration. If necessary, the final library was purified and concentrated by using CleanNGS beads. The quality and integrity of the library were confirmed using the Agilent 2200 TapeStation with D1000 ScreenTapes (Agilent Technologies, Santa Clara, California, USA).

Sequences were generated at the Berlin Centre for Genomics in Biodiversity Research (BeGenDiv) in two runs on the Illumina MiSeq platform (Illumina, San Diego, California, USA) using v3 chemistry with 600 cycles. The quality of the generated reads was evaluated using FastQC v.0.11.9 and multiqc. The remaining adapter sequences were removed using cutadapt (version 2.8).

Sequencing read processing from quality control to taxonomic assignment was performed using the R package “dada2” (Callahan et al., 2016). We assigned taxonomy to the inferred Amplified Sequence Variants (ASVs) up to species level based on the reference database provided by BeGenDiv (Heller et al., 2018). Taxonomy was assigned based on the single best hit or a last common ancestor (in case of multiple best hits), with 50 out of 100 bootstrap replicates as the minimum bootstrap confidence for assigning a taxonomic level. For post‐sequencing removal of reads caused by contamination, we used the R package “microDecon” (McKnight et al., 2019), which uses the proportions of ASVs in blank samples (negative controls) to systematically identify and remove contaminant reads from the metabarcoding data set. Afterward, we summed up reads for pseudo‐biological replicates and removed reads which were only present in one of two technical replicates to further increase the power and quality of our data set.

We restricted our dataset to results of sequences on the species level and deleted the finding of bat sequences (*Myotis daubentoniid* and *Pipistrellus pygmaeus*) in the gut content of seven fish individuals, as we assumed that this was not the results of selective feeding on the bats, but instead the incorporation of bat feces. All species identified in the gut content were classified into the categories “aquatic” and “terrestrial,” corresponding to their dominant life phases and we counted the number of ingested terrestrial species for each fish individual.

### 2.4 Stable isotope analysis

Analyses of stable isotopes of hydrogen (δ^2^H) of fish muscle tissue (bleak (*Alburnus alburnus*): *N* = 27, perch (*Perca fluviatilis*): *N* = 13, roach (*Rutilus rutilus*): *N* = 26), seston (*N* = 6), and terrestrial insects (*N* = 12) samples were conducted at the Leibniz‐Institute for Zoo‐and Wildlife Research Berlin. To remove contaminants, each insect sample was washed using a 2:1 chloroform: methanol solution for 24 h. Fish muscle samples were fat‐extracted using a Soxtherm extractor (C. Gerhardt GmbH & Co. KG, Königswinter, Germany). After being dried in an oven at 50°C for 72 hours, sample aliquots of 0.35 ± 0.10 mg were placed into silver capsules (IVA Analysentechnik, Meerbusch, Germany). Non‐exchangeable hydrogen isotope ratios were measured using a High Temperature Conversion Elemental Analyzer (Thermo Fischer Scientific Inc., Waltham, USA) connected to an online temperature‐controlled vacuum‐equilibration autosampler Uni‐Prep (EuroVector, Pavia, Italy) and coupled to a continuous‐flow isotope‐ratio mass spectrometer (Delta V Advantage; Thermo Fischer Scientific Inc.). Measurements were determined using a comparative equilibration method (Wassenaar & Hobson, 2003). Samples and standards were loaded into the autosampler at 60°C (Soto et al., 2017, 2019). After flushing with helium and evacuating the carousel, 20 μL of water of known isotopic composition (δ^2^H = −54.42‰) was injected trough the Uni‐Prep septum for equilibration (1 h) (Soto et al., 2017, 2019). To ensure that a similar H exchangeability among standards and samples, samples were measured together with in‐house standards, previously calibrated against international reference materials. Stable hydrogen isotope ratios were expressed as deviations from the international standard V‐SMOW. Precision of δ^2^H measurements was always better than 1.1‰ (1 SD). The hydrogen exchangeability of muscle, chitin, and plant material was previously determined applying the two‐water equilibration approach and using two isotopically distinct waters (δ^2^H = −54.42‰ and −427.50‰) on three in‐house muscle standards, two in‐house chitin standards, and algal test material. The fraction of exchangeable hydrogen (ƒ_ex_) of each type of material was calculated following the equation by Soto et al. (2017):

$$f_{\mathrm{ex}}=\frac{\delta^{2}H_{\mathrm{mat}-\mathrm{EW}}-\delta^{2}H_{\mathrm{mat}-\mathrm{DW}}}{\delta^{2}H_{w-\mathrm{EW}}-\delta^{2}H_{w-\mathrm{DW}}}$$

where $\delta^{2}H_{\mathrm{mat}}$ are the δ^2^H the isotope values of standards/test materials measured after the equilibration with enriched water (EW) and depleted water (DW), respectively, and $\delta^{2}H_{w}$ are the isotope values of the two equilibration waters. The resulting ƒ_ex_ values for muscle, chitin, and algae were calculated to be 2.79%, 1.80%, and 2.10%, respectively. These values were applied to this study to estimate the non‐exchangeable δ^2^H of the samples (δ^2^H_n_) using the following equation:

$$\delta^{2}H_{\mathrm{tot}}=f_{\mathrm{ex}}\times \delta^{2}H_{\mathrm{ex}}+1-f_{\mathrm{ex}}\times \delta^{2}H_{n}$$

where $\delta^{2}H_{\mathrm{tot}}$ is the measured δ^2^H of the sample and $\delta^{2}H_{\mathrm{ex}}$ is the isotopic composition of the exchangeable hydrogen.

Muscle samples were measured in sequence with three in‐house muscle standards: MUS1 (δ^2^H = −107.60‰), MUS2 (δ^2^H = −105.80‰), MUS3 (δ^2^H = −60.38‰). The rest of the samples were measured in sequence with two in‐house chitin standards: Chitin H (δ^2^H = −40.32‰) and Chitin K (δ^2^H = −21.76‰). All in‐house standards were previously calibrated against international reference materials (USGS42, CBS, KHS, (Soto et al., 2017)). Stable hydrogen isotope ratios were expressed as deviations from the international standard V‐SMOW. Precision of δ^2^H measurements was always better than 1.1‰ (1 SD).

### 2.5 Terrestrial index modeling

Prior to estimating the contribution of terrestrial species to the diet of individual fish (i.e., terrestrial index), we corrected for the influence of environmental (or dietary) water on the δ^2^H of fish (Solomon et al., 2009) and followed the approach outlined in Vander Zanden et al. (2016):

$$\omega_{\text{compound}}=1-{\left(1-\omega \right)}^{\mathrm{TL}-1}$$

where $\omega_{\text{compound}}$ is the proportion of water δ^2^H in δ^2^H values of fish consumers, ω is the proportion of water δ^2^H entering the consumer. For ω, we assumed a value of 0.28 as suggested by Soto et al. (2019). We did not estimate the trophic level (TL) of the fish, but assumed a theoretical level of 2.5, as we analyzed omnivorous species and individuals (Bíró & Muskó, 1995; Latorre et al., 2023; Marklund et al., 2019; Persson, 1983).

In a second step, we modeled δ^2^H values for fish consumers either obtaining their unexchangeable hydrogen either entirely from aquatic, or terrestrial resources:

$$\delta^{2}H_{C\;100\%\text{aquatic}}=\left(\omega_{\text{compound}}\times \delta^{2}H_{\text{water}}\right)+\left(1-\omega_{\text{compound}}\right)\times \delta^{2}H_{\text{aquatic}}$$

$$\delta^{2}H_{C\;100\%\text{terrestrial}}=\left(\omega_{\text{compound}}\times \delta^{2}H_{\text{water}}\right)+\left(1-\omega_{\text{compound}}\right)\times \delta^{2}H_{\text{terrestrial}}$$

$\delta^{2}H_{\text{water}}$ is the δ^2^H of the river water. We did not collect water samples ourselves, but instead used a mean value (−55.06‰) from Chen et al. (2023), who collected river water in Spremberg and Zerre in June and December 2021. For $\delta^{2}H_{\text{aquatic}}$, we used the mean value of seston (from locations downstream and upstream of the dam respectively), whereas for $\delta^{2}H_{\text{terrestrial}}$, we used the mean value of the terrestrial insects (from locations downstream and upstream of the dam respectively). Values of terrestrial insects were corrected for the influence of dietary water and trophic compounding (assuming a trophic level of 2), similarly as described above for fish consumers.

The terrestrial index for individual fish was modeled, using a modified two‐end members mixing model similar to the allochthony index from Keva et al. (2022).

$$\text{Terrestrial index}=\frac{\left(\delta^{2}H_{C\;}-\delta^{2}H_{C\;100\%\text{aquatic}}\right)}{\left(\delta^{2}H_{C\;100\%\text{terrestrial}}-\delta^{2}H_{C\;100\%\text{aquatic}}\right)}$$

$\delta^{2}H_{C}$ is the measured value of the individual fish consumer. The terrestrial index ranges from 0 to 1, with high values indicating the incorporation of hydrogen from terrestrial insects, while low values indicate the incorporation of hydrogen from aquatic insects.

### 2.6 Statistical analyses

Data analyses were conducted in R version 4.1.0 (R Core Team, 2022) and R Studio 1.4.1717. For the analyses of fish community composition, we removed all fish species with an abundance ≤0.1% and we focused on the remaining 15 species. We used the vegan package and conducted Analysis of similarities (ANOSIM) on the Bray‐Curtis dissimilarly measure (9999 permutations) to determine the significance of differences among communities of benthic invertebrates and fish upstream and downstream of the dam. We further used non‐parametric Mann–Whitney *U* Tests to compare the total abundance of benthic invertebrates, CPUE and BPUE of fish, and condition factor of pike upstream and downstream of the dam. We used Mann–Whitney *U* Tests to compare the number of terrestrial insects in the gut, as well as the terrestrial index for the three most abundant fish species (i.e. roach, bleak, perch) upstream and downstream of the dam.

## 3 RESULTS

### 3.1 Macroinvertebrate and fish community

**FIGURE 2:**
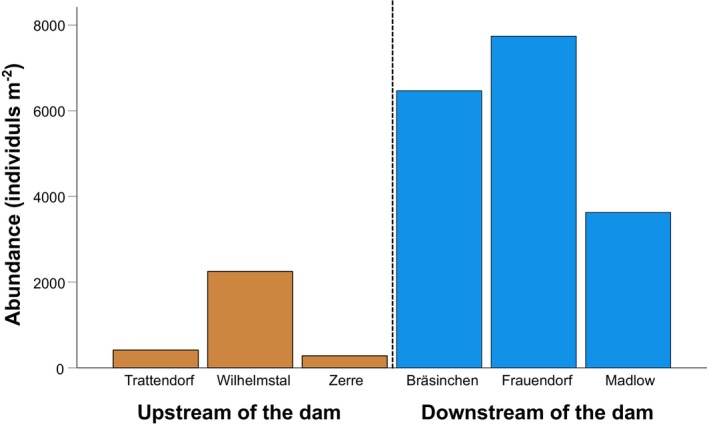
Abundance of benthic macroinvertebrates (individuals m^−2^) collected at the three locations upstream (i.e., high iron concentrations) and the three locations downstream (i.e., low iron concentrations) of the dam. See Appendix S1 for a list of invertebrate taxa found at each site.

While Chironomidae were the most abundant taxa in the invertebrate samples of sites located upstream of the dam, Bivalvia and Gastropoda dominated the community composition upstream of the dam (Appendix S1). However, ANOSIM revealed that the composition of community structure of benthic invertebrates upstream and downstream of the dam was not significantly different. Total abundance was significantly higher at locations downstream of the dam (*Z* = 3.86, *p* = .0495; 5945 individuals m^−2^ ± 2106, mean ± SD) compared to locations upstream of the dam (982 individuals m^−2^ ± 1100; Figure 2).

**FIGURE 3:**
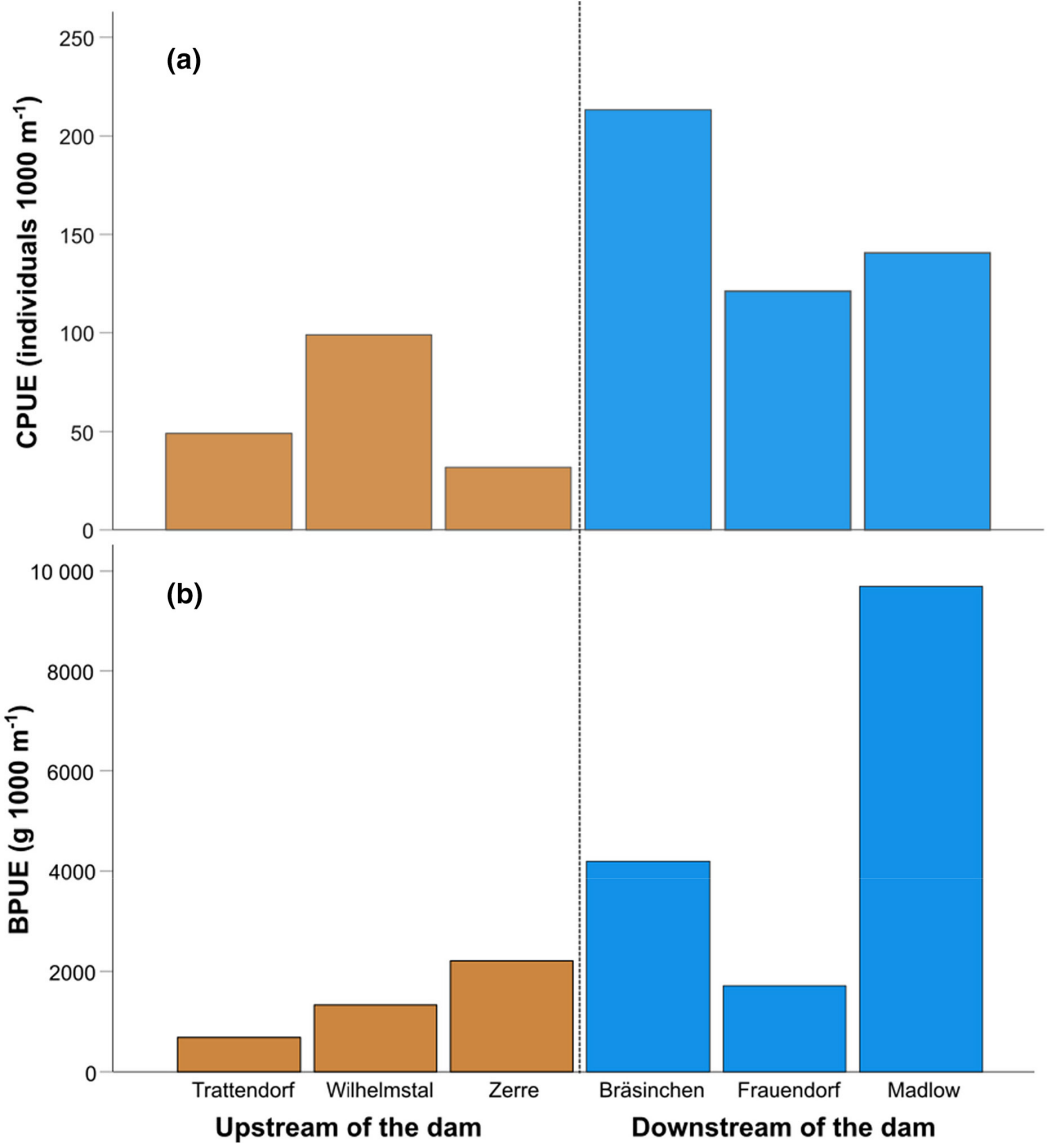
(a) Abundance (represented as catch per unit effort, CPUE, individuals 1000 m^−1^) and (b) biomass of fish species (represented as biomass per unit effort, BPUE, g 1000 m^−1^) collected at the three locations upstream (i.e., high iron concentrations) and the three locations downstream (i.e., low iron concentrations) of the dam. See Appendix S2 for a list of species abundances at each site.

We caught 20 fish species in total (Appendix S2). The three most abundant fish species were roach, bleak, and perch (37.6%, 17.5%, and 15.7% of total abundance, respectively; Appendix S2). ANOSIM indicated that the fish community composition upstream and downstream of the dam was not significantly different, neither when accounting abundances (i.e., CPUE), nor biomass data (i.e., BPUE). However, total CPUE of all fishes was significantly higher in locations downstream of the dam (*Z* = 3.86, *p* = .0495; 158 individuals 1000 m^−1^ ± 49) compared to locations upstream of the dam (60 individuals 1000 m^−1^ ± 35; Figure 3), but BPUE was not significantly higher.

### 3.2 Condition factor of pike

In total, we caught 55 pike, 31 individuals upstream and 24 individuals downstream of the dam. From scale annuli, we were able to clearly identify age‐0 pike (*N* = 35), but for other cohorts, age identification was not so clear and we pooled all individuals < age‐0. Condition factor of age‐0 pike individuals was significantly higher downstream of the dam (0.64 ± 0.12, average ± SD), compared to individuals upstream of the dam (0.54 ± 0.05; *Z* = 11.33, *p* < .0001). Similarly, condition factor of adult pike individuals > age‐0 was significantly higher downstream of the dam (0.68 ± 0.08, average ± SD) compared to individuals upstream of the dam (0.60 ± 0.04; *Z* = 6.325, *p* = .012).

### 3.3 Metabarcoding

Unfortunately, some problems during the PCR procedure in the lab appeared that resulted in fewer fish individuals with a sequenced gut content. We identified 32 aquatic and 17 terrestrial species in the gut content of the 31 fish individuals analyzed (Appendix S1). Fish individuals feed on 1–8 different species and perch, irrespective of the location, had the most diverse diet (5 ± 3 species), in contrast to roach (3 ± 2 species), and bleak (2 ± 1 species) (Table 2). No terrestrial species were identified in the gut content of fish caught downstream of the dam and no terrestrial species were identified in the gut content of roaches upstream of the dam either, thus, not allowing any statistical comparison (Table 2). However, in locations upstream of the dam, terrestrial species were ingested by bleak (1 species ±1) and perch (4 species ±1) and this result was significant for the latter species (*Z* = 6.137, *p* = .013, Table 2).

**TABLE 2:** Overview on the number of ingested aquatic and terrestrial species identified using metabarcoding of gut content of perch, bleak, and roach caught in river sections (a) upstream of the dam (i.e., high iron concentrations); and (b) downstream of the dam (i.e. low iron concentrations). See Appendix S1 for an extended list of the different species.

	Number of species identified (average ± standard deviation)		
	Perch	Bleak	Roach
*(a) Upstream of the dam*			
Aquatic species	3 ± 1	1 ± 1	2 ± 1
Terrestrial species	4 ± 1	1 ± 1	0
*(b) Downstream of the dam*			
Aquatic species	3 ± 3	2 ± 1	4 ± 2
Terrestrial species	0	0	0

### 3.4 Stable isotope analysis and terrestrial index

**FIGURE 4:**
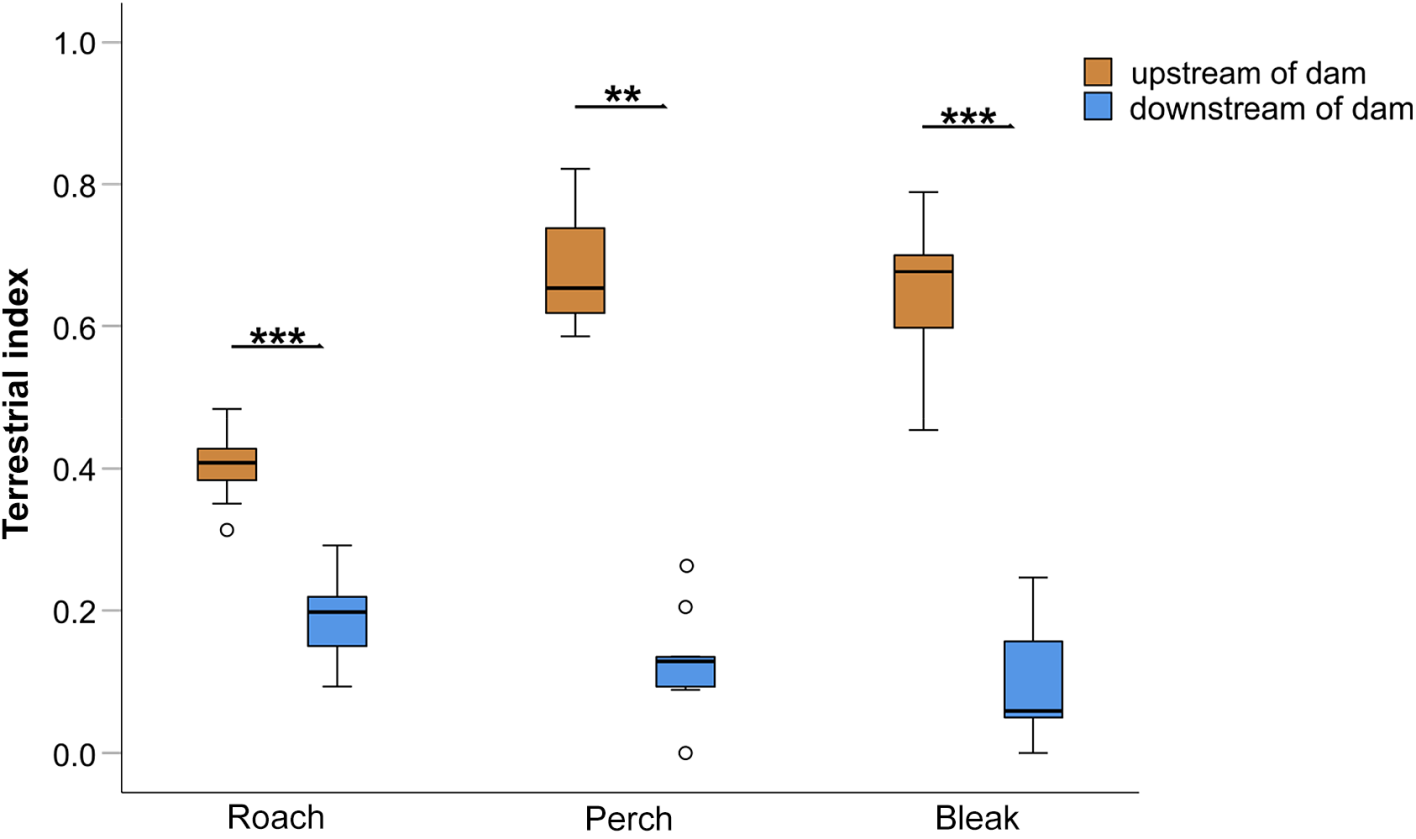
Contribution of aquatic and terrestrial resources to the diet of the three dominant fish species (roach, perch, and bleak), as depicted by the terrestrial index of fishes. A higher index indicates higher contribution of terrestrial resources to the diet. Boxplots indicate medians, with whiskers expanding to the 25th and 75th percentile. Dots denote outliers and asterisks level of significance (***p ≤ .001; **p ≤ .01).

Generally, seston samples were more depleted in deuterium, and thus had lower δ^2^H values compared to terrestrial insects (Appendix S2). Terrestrial index (i.e., the contribution of terrestrial resources to the diet of the consumer) was significantly higher for roach (*Z* = 16.25; *p* < .001; 0.41 ± 0.05), perch (*Z* = 7.71; *p* = .005; 0.68 ± 0.10), and bleak (*Z* = 21.39; *p* = <.001; 0.64 ± 0.10) caught upstream of the dam, compared with individuals of the respective species downstream of the dam (roach: 0.19 ± 0.06, perch: 0.13 ± 0.07; bleak: 0.10 ± 0.08; Figure 4).

## 4 DISCUSSION

In this study, we demonstrate that iron flocculation resulting from open‐cast lignite mining has the ability to restructure the lotic food web, including the abundance of benthic invertebrates and fishes, growth of young‐of‐the‐year top predators and the strength of the linkage to the adjacent terrestrial ecosystem. While several studies have shown the consequences of iron concentrations for single species or specific communities, our study is among the first that integrates consequences to stream functioning and structure, that is, the animal‐mediated fluxes occurring across the land‐water interface.

Fluxes from land to water occur primarily in the appearance of terrestrial carbon from plants, either in the particulate form as plant litter or in dissolved form, mostly as humic substances. Several studies could demonstrate that terrestrial carbon has the ability to fuel aquatic food webs and can be traced up to the highest trophic levels (Karlsson et al., 2012; Scharnweber et al., 2014; Tanentzap et al., 2014). However, another pathway of terrestrial particulate carbon into aquatic systems, especially important for lotic systems, is the one via terrestrial insects falling on the water surface and being consumed by freshwater fish (Baxter et al., 2005). In the famous greenhouse experiments of Nakano et al. (1999), terrestrial insects were prevented from falling into a forest stream, and the strong impact of these pathways could be demonstrated, being able to shape the feeding ecology (Fausch et al., 1997) and overall fish biomass (Kawaguchi & Nakano, 2001).

In the present study, we demonstrate how the human impact on land use, more precisely via the contamination of iron resulting from lignite mining is able to alter these linkages and shape fish communities. Generally, fish abundance and biomass are known to be low in lotic systems characterized by high iron concentrations (Amisah & Cowx, 2000; Vuori, 1995), and our results are in line with these previous findings. The low abundance of benthic invertebrates as food, as demonstrated in this study, might play a crucial role in this correlation. Such low availability of aquatic prey at high iron section of the Spree River might force some fish species to integrate terrestrial insects into their diet. Here, we could demonstrate this switch using a combination of techniques illustrating not only the short‐term (metabarcoding of gut content) but also long‐term (stable isotopes of hydrogen) resource use. Indicated by the DNA sequences from metabarcoding, perch and bleak caught upstream of the dam had a higher number of terrestrial species in their gut content, but this result was not significant for roaches. While perch and roach are known to usually ingests only minor proportions of terrestrial insects (e.g., Marklund et al., 2019; Persson, 1983; Svanbäck & Bolnick, 2007), bleak has an upward‐facing snout showing the adaptions feeding on insects from the water surface (Bíró & Muskó, 1995; Latorre et al., 2023). The metabarcoding approach does not allow for a quantitative assessment of the ingested prey, as biomass estimates are not possible. Furthermore, we cannot rule out the possibility that DNA of terrestrial insects might have been ingested from upstream decomposing individuals in the river water. However, the pattern was further confirmed by the stable isotope data, and for perch, bleak and also roach, the incorporation of terrestrial resources to the diet increased significantly from 14% on average at low iron sections to 55% on average. Terrestrial species identified in the gut content of the fish included a wide range of species and taxa, including earthworms, ants or flying insects and most identified species (except for two species) appeared only once in the entire dataset. Fish that incorporate terrestrial species into their diet may therefore not specialize on specific prey, but might respond rather opportunistically to organisms that end up on the stream surface.

Along the same line, Soto et al. (2019) reported high contributions from allochthonous plants to the fish community of the Congo River, which is characterized by high sediment loads. This similarity suggests the increasing importance of allochthonous sources under high‐turbidity conditions that may suppress the autochthonous river production.

Significant differences between the terrestrial reliance of fish individuals upstream and downstream of the dam were pronounced, as demonstrated by the two methods integrating different time scales. However, some uncertainty in the absolute values of the terrestrial contributions has to be considered. When correction for the proportion of water δ^2^H entering the consumer, we applied a correction factor of 0.28, as suggested by Soto et al. (2019). However, this correction factor is still under debate and the literature also shows that the value of ω can be assumed as 0.17 (Solomon et al., 2009) or averaging 0.25–0.30 (Brett et al., 2018). Furthermore, while correcting for the trophic compounding effect, we did not estimate the trophic level of the aquatic consumers individually. The outputs of the mixing models are very dependent on these assumptions, thus making it difficult to compare the obtained estimates to previous reported terrestrial contributions from other systems. The results of the rather simple two‐end members mixing model should thus be considered as a proof of concept of the terrestrial linkage to the aquatic food web.

Such diet shifts toward a higher integration of terrestrial insects was also reported from brook and brown trout (*Salvelinus fontinalis* and *Salmo trutta*) that compensated for the low availability of aquatic prey in stream sections of high heavy metal concentrations (Kraus et al., 2016). However, the feeding on terrestrial organisms may come at a cost. First, fish individuals feeding on the water surface might be more vulnerable to attacks from aerial predators. In addition, aquatic resources are generally rich in polyunsatuarted fatty acids (PUFAs), while terrestrial insects are characterized by low concentrations of these important biomolecules (Twining et al., 2016, 2021). Thus, fish individuals feeding to a high degree on terrestrial insects and therefore facing a diet of lower quality when it comes to PUFA content, might have to internally synthesize PUFAs to meet the physiological need (Scharnweber et al., 2021), which could potentially affect growth and fitness (Scharnweber & Gårdmark, 2020; Závorka et al., 2022).

A reduction of energy at the base of the food web was further translated up the food chain, thus it most likely reduced the condition in juvenile and adult pike, which is the most abundant top predator in this system. Juvenile pike in their first year caught in high‐iron sections were on average 9.9 cm smaller compared to juveniles caught in sections of clear water (data not shown). Turbidity in the high‐iron sections may affect the foraging abilities of pike as a visual predator by altering the reactive distance to the prey fish, with consequences for attack rates and the amount of consumed prey (Jacobsen & Engström‐Öst, 2018). However, pike might compensate low visibility by other sensory modes, such as the ones from the lateral line (Raat, 1988). Instead, it can reasonably be assumed that the differences in total length of juvenile pike is rather caused by the overall low availability of food (i.e., low abundances of prey fish) and likely the limited aquatic vegetation, which is important as habitat for the juvenile pike (Jacobsen & Engström‐Öst, 2018). Unfortunately, low sample sizes of adult pike individuals did not allow further comparisons of cohorts, but it can be assumed that pike of all cohorts living in high‐iron sections of the Spree River have an overall lower fitness. Our results thus highlight the broad impact of iron load on each level of the aquatic food web. The results presented here, however, represent a single time point of the year. We would like to emphasize that longer studies, including the seasonal and temporal variances of abundances and fish resource use are needed to draw more general conclusions on the post‐mining effects on the lotic food webs.

Besides the overall low availability of aquatic prey, other factors may add to the detrimental effects of iron (III) on fish communities. For example, iron could directly impact fish, as precipitates can clog gills and eggs (Andersson & Nyberg, 1984; Gerhardt, 1992), but further indirect effects are possible. The growth of submerged macrophytes, for instance, can be hampered under brown water conditions (Blanchet et al., 2022). Macrophytes provide a complex habitat, favouring fish and invertebrate diversity and richness simultaneously (Soukup et al., 2022). Certainly, more studies are needed to understand the underlying and potentially multifactorial causes for the low fish abundance found in this study.

Intensive land use from mining activities may not be the only human‐induced perturbation acting on the river food web, as multiple stressors often act jointly (Sabater et al., 2019). The modification of the river flow via the building of dams poses one of the biggest impacts on rivers across the globe (e.g., Dudgeon et al., 2006; Schmutz & Moog, 2018). The disruption of the river flow by damming and the formation of a reservoir is a drastic process which will alter many aspects of the ecosystems, for example, fish migration and the transport of nutrients. An additional factor may occur from the modification of the downstream thermal regime, which depends on the mode of operation and specific mechanism of the water release from the reservoir (Olden & Naiman, 2010). While many large dams may release cold water from below the thermocline of the reservoir, more shallow reservoirs may alter the downstream thermal regime by warming with profound impact on metabolic rates, physiology, and life‐history traits of aquatic species (Lessard & Hayes, 2003; Olden & Naiman, 2010). The rather shallow Spremberg reservoir releases the water from the bottom, but the reservoir itself is usually not stratified (data not shown). In the study presented here, the river water downstream of the dam was generally warmer during summertime but colder during wintertime (Appendix S3). This indicates that the Spremberg Reservoir severely alters the thermal regime further downstream. This pattern could potentially affect the abundance of invertebrates and fish and further the growth of pike. The study setup does not allow for the differentiation between the effects of iron flocculation and the changes of the water temperature downstream of the dam and further studies are needed to investigate the cumulative stressor of human‐induced perturbations on our river systems.

## 5 CONCLUSION

In this study, we show how land use, that is, lignite mining activities and the resulting iron flocculation in lotic systems may decrease benthic invertebrate and fish biomass. In addition, we were able to demonstrate the potential of mining activities to restructure aquatic food webs, forcing the fish community to an increased reliance on terrestrial insects. Therefore, mining activities represent an environmental perturbation that can alter the flow of energy between aquatic and terrestrial systems. Another human‐induced impact that could potentially act in a similar way is the one of the installations of artificial light near a waterbody which could attract more insects to be closer to the water (Perkin et al., 2011), but empirical evidence is missing. We therefore agree with Soininen et al. (2015) and argue to incorporate a holistic and unifying view of ecology on the landscape level when considering the effects of human‐induced environmental change on our ecosystems.

## AUTHOR CONTRIBUTIONS

**Kristin Scharnweber:** Conceptualization (equal); data curation (equal); formal analysis (equal); funding acquisition (equal); investigation (lead); methodology (lead); project administration (lead); resources (equal); software (equal); supervision (equal); validation (equal); visualization (equal); writing – original draft (lead); writing – review and editing (equal). **Carolin Scholz:** Data curation (equal); formal analysis (equal); methodology (equal). **Victor Schippenbeil:** Formal analysis (equal). **Stefania Milano:** Formal analysis (equal). **Daniel Hühn:** Conceptualization (equal); formal analysis (equal); funding acquisition (equal); methodology (equal); supervision (equal); validation (equal); visualization (equal); writing – review and editing (equal).

## FUNDING INFORMATION

This study was financed by the research training group GRK 2118/2 “BioMove” of the German Research Foundation (DFG) and the Fischereiabgabe Brandenburg.

## CONFLICT OF INTEREST STATEMENT

The authors declare that they do not have any conflict of interest.

## ETHICAL APPROVAL

All applicable institutional and national guidelines for the care and use of animals were followed.

## Supporting information


Appendix S1

## Data Availability

Raw data and R scripts will be available on the openly accessible repository Zenodo (10.5281/zenodo.10692513).

## References

[ece311111-bib-0001] Amisah, S. , & Cowx, I. G. (2000). Impacts of abandoned mine and industrial discharges on fish abundance and macroinvertebrate diversity of the upper River Don in South Yorkshire, UK. Journal of Freshwater Ecology, 15, 237–250.

[ece311111-bib-0002] Amundsen, P. A. , & Sánchez‐Hernández, J. (2019). Feeding studies take guts – critical review and recommendations of methods for stomach contents analysis in fish. Journal of Fish Biology, 95, 1364–1373. 31589769 10.1111/jfb.14151

[ece311111-bib-0003] Andersson, P. , & Nyberg, P. (1984). Experiments with brown trout (*Salmo trutta* L.) during spring in mountain streams at low pH and elevated levels of iron, manganese and aluminium. Report‐Institute of Freshwater Research, Drottningholm, 61, 34–47.

[ece311111-bib-0004] Bartels, P. , Cucherousset, J. , Steger, K. , Eklöv, P. , Tranvik, L. J. , & Hillebrand, H. (2012). Reciprocal subsidies between freshwater and terrestrial ecosystems structure consumer resource dynamics. Ecology, 93, 1173–1182. 22764503 10.1890/11-1210.1

[ece311111-bib-0005] Baxter, C. V. , Fausch, K. D. , & Saunders, W. C. (2005). Tangled webs: Reciprocal flows of invertebrate prey link streams and riparian zones. Freshwater Biology, 50, 201–220.

[ece311111-bib-0006] Benthaus, F.‐C. , & Totsche, O. (2015). The groundwater raise in lignite mining induced areas in Lusetia – Actions taken to reduce the follows ups. Mining Reports, 06, 522–530.

[ece311111-bib-0007] Bíró, P. , & Muskó, I. B. (1995). Population dynamics and food of bleak (*Alburnus alburnus* L) in the littoral zone of Lake Balaton, Hungary. Hydrobiologia, 310, 139–149.

[ece311111-bib-0008] Blanchet, C. C. , Arzel, C. , Davranche, A. , Kahilainen, K. K. , Secondi, J. , Taipale, S. , Lindberg, H. , Loehr, J. , Manninen‐Johansen, S. , Sundell, J. , Maanan, M. , & Nummi, P. (2022). Ecology and extent of freshwater browning‐what we know and what should be studied next in the context of global change. Science of the Total Environment, 812, 152420. 34953836 10.1016/j.scitotenv.2021.152420

[ece311111-bib-0009] Boecklen, W. J. , Yarnes, C. T. , Cook, B. A. , & James, A. C. (2011). On the use of stable isotopes in trophic ecology. Annual Review of Ecology, Evolution, and Systematics, 42, 411–440.

[ece311111-bib-0010] Brett, M. T. , Holtgrieve, G. W. , & Schindler, D. E. (2018). An assessment of assumptions and uncertainty in deuterium‐based estimates of terrestrial subsidies to aquatic consumers. Ecology, 99, 1073–1088. 29714826 10.1002/ecy.2211

[ece311111-bib-0011] Byrne, P. , Wood, P. J. , & Reid, I. (2012). The impairment of river systems by metal mine contamination: A review including remediation options. Critical Reviews in Environmental Science and Technology, 42, 2017–2077.

[ece311111-bib-0012] Cadmus, P. , Clements, W. H. , Williamson, J. L. , Ranville, J. F. , Meyer, J. S. , & Gines, M. J. G. (2016). The use of field and mesocosm experiments to quantify effects of physical and chemical stressors in mining‐contaminated streams. Environmental Science & Technology, 50, 7825–7833. 27362637 10.1021/acs.est.6b01911 PMC5744682

[ece311111-bib-0013] Callahan, B. J. , McMurdie, P. J. , Rosen, M. J. , Han, A. W. , Johnson, A. J. A. , & Holmes, S. P. (2016). DADA2: High‐resolution sample inference from Illumina amplicon data. Nature Methods, 13, 581–583. 27214047 10.1038/nmeth.3869 PMC4927377

[ece311111-bib-0014] Chen, K. , Tetzlaff, D. , Goldhammer, T. , Freymueller, J. , Wu, S. , Schmidt, A. , Liu, G. , Venohr, M. , & Soulsby, C. (2023). Synoptic water isotope surveys to understand the hydrology of large intensively managed catchments. Journal of Hydrology, 623, 129817.

[ece311111-bib-0015] Cid, N. , Eros, T. , Heino, J. , Singer, G. , Jähnig, S. C. , Canedo‐Arguelles, M. , Bonada, N. , Sarremejane, R. , Mykrä, H. , Sandin, L. , Paloniemi, R. , Varumo, L. , & Datry, T. (2022). From meta‐system theory to the sustainable management of rivers in the Anthropocene. Frontiers in Ecology and the Environment, 20, 49–57. 35873359 10.1002/fee.2417 PMC9292669

[ece311111-bib-0016] Doucett, R. R. , Marks, J. C. , Blinn, D. W. , Caron, M. , & Hungate, B. A. (2007). Measuring terrestrial subsidies to aquatic food webs using stable isotopes of hydrogen. Ecology, 88, 1587–1592. 17601150 10.1890/06-1184

[ece311111-bib-0017] Dudgeon, D. (2019). Multiple threats imperil freshwater biodiversity in the Anthropocene. Current Biology, 29, R960–R967. 31593677 10.1016/j.cub.2019.08.002

[ece311111-bib-0018] Dudgeon, D. , Arthington, A. H. , Gessner, M. O. , Kawabata, Z. I. , Knowler, D. J. , Leveque, C. , Naiman, R. J. , Prieur‐Richard, A. H. , Soto, D. , Stiassny, M. L. J. , & Sullivan, C. A. (2006). Freshwater biodiversity: Importance, threats, status and conservation challenges. Biological Reviews, 81, 163–182. 16336747 10.1017/S1464793105006950

[ece311111-bib-0019] Edwards, E. D. , & Huryn, A. D. (1996). Effect of riparian land use on contributions of terrestrial invertebrates to streams. Hydrobiologia, 337, 151–159.

[ece311111-bib-0020] Fausch, K. D. , Nakano, S. , & Kitano, S. (1997). Experimentally induced foraging mode shift by sympatric charrs in a Japanese mountain stream. Behavioral Ecology, 8, 414–420.

[ece311111-bib-0021] Foley, J. A. , DeFries, R. , Asner, G. P. , Barford, C. , Bonan, G. , Carpenter, S. R. , Chapin, F. S. , Coe, M. T. , Daily, G. C. , Gibbs, H. K. , Helkowski, J. H. , Holloway, T. , Howard, E. A. , Kucharik, C. J. , Monfreda, C. , Patz, J. A. , Prentice, I. C. , Ramankutty, N. , & Snyder, P. K. (2005). Global consequences of land use. Science, 309, 570–574. 16040698 10.1126/science.1111772

[ece311111-bib-0022] Friedland, G. , Grüneberg, B. , & Hupfer, M. (2021). Geochemical signatures of lignite mining products in sediments downstream a fluvial‐lacustrine system. Science of the Total Environment, 760, 143942. 33348154 10.1016/j.scitotenv.2020.143942

[ece311111-bib-0023] Galan, M. , Pons, J. B. , Tournayre, O. , Pierre, E. , Leuchtmann, M. , Pontier, D. , & Charbonnel, N. (2018). Metabarcoding for the parallel identification of several hundred predators and their prey: Application to bat species diet analysis. Molecular Ecology Resources, 18, 474–489. 29288544 10.1111/1755-0998.12749

[ece311111-bib-0024] Gerhardt, A. (1992). Effects of subacute doses of iron (Fe) on *Leptophlebia marginata* (Insecta, Ephemeroptera). Freshwater Biology, 27, 79–84.

[ece311111-bib-0025] Gray, N. F. (1997). Environmental impact and remediation of acid mine drainage: A management problem. Environmental Geology, 30, 62–71.

[ece311111-bib-0026] Grey, J. (2006). The use of stable isotope analyses in freshwater ecology: Current awareness. Polish Journal of Ecology, 54, 563–584.

[ece311111-bib-0027] Heller, P. , Casaletto, J. , Ruiz, G. , & Geller, J. (2018). A database of metazoan cytochrome c oxidase subunit I gene sequences derived from gen Bank with CO‐ARBitrator. Scientific Data, 5, 1–7. 30084847 10.1038/sdata.2018.156 PMC6080493

[ece311111-bib-0028] Htun‐Han, M. (1978). The reproductive biology of the dab *Limanda limanada* (L.) in the North Sea: Gonadosomatic index, hepatosomatic index and condition factor. Journal of Fish Biology, 13, 351–377.

[ece311111-bib-0029] Jacobsen, L. , & Engström‐Öst, J. (2018). Coping with environments, vegetation, turbidity, and abiotics. In C. Skov & P. Nilsson (Eds.), Biology and ecology of pike. CRC Press, Taylor and Francis Group.

[ece311111-bib-0030] Karlsson, J. , Berggren, M. , Ask, J. , Bystrom, P. , Jonsson, A. , Laudon, H. , & Jansson, M. (2012). Terrestrial organic matter support of lake food webs: Evidence from lake metabolism and stable hydrogen isotopes of consumers. Limnology and Oceanography, 57, 1042–1048.

[ece311111-bib-0031] Kawaguchi, Y. , & Nakano, S. (2001). Contribution of terrestrial invertebrates to the annual resource budget for salmonids in forest and grassland reaches of a headwater stream. Freshwater Biology, 46, 303–316.

[ece311111-bib-0032] Keva, O. , Kiljunen, M. , Hamalainen, H. , Jones, R. I. , Kahilainen, K. K. , Kankaala, P. , Laine, M. B. , Schilder, J. , Strandberg, U. , Vesterinen, J. , & Taipale, S. J. (2022). Allochthony, fatty acid and mercury trends in muscle of Eurasian perch (*Perca fluviatilis*) along boreal environmental gradients. Science of the Total Environment, 838, 155982. 35588838 10.1016/j.scitotenv.2022.155982

[ece311111-bib-0033] Kraus, J. M. , Pomeranz, J. F. , Todd, A. S. , Walters, D. M. , Schmidt, T. S. , & Wanty, R. B. (2016). Aquatic pollution increases use of terrestrial prey subsidies by stream fish. Journal of Applied Ecology, 53, 44–53.

[ece311111-bib-0034] Krümmelbein, J. , Bens, O. , Raab, T. , & Naeth, M. A. (2012). A history of lignite coal mining and reclamation practices in Lusatia, eastern Germany. Canadian Journal of Soil Science, 92, 53–66.

[ece311111-bib-0035] Larsen, S. , Muehlbauer, J. D. , & Marti, E. (2016). Resource subsidies between stream and terrestrial ecosystems under global change. Global Change Biology, 22, 2489–2504. 26649817 10.1111/gcb.13182

[ece311111-bib-0036] Latorre, D. , Masó, G. , Cano‐Barbacil, C. , Zamora‐Marin, J. M. , Almeida, D. , Vilizzi, L. , Britton, J. R. , Cruz, A. , Fernández‐Delgado, C. , González‐Rojas, A. G. , Miranda, R. , Rubio‐Gracia, F. , Tarkan, A. S. , Torralva, M. , Vila‐Gispert, A. , Copp, G. H. , & Ribeiro, F. (2023). A review and meta‐analysis of the environmental biology of bleak *Alburnus alburnus* in its native and introduced ranges, with reflections on its invasiveness. Reviews in Fish Biology and Fisheries, 33, 931–975.

[ece311111-bib-0037] Lessard, J. L. , & Hayes, D. B. (2003). Effects of elevated water temperature on fish and macroinvertebrate communities below small dams. River Research and Applications, 19, 721–732.

[ece311111-bib-0038] Letterman, R. D. , & Mitsch, W. J. (1978). Impact of mine drainage on a mountain stream in Pennsylvania. Environmental Pollution, 17, 53–73.

[ece311111-bib-0039] Manko, P. (2016). Stomach content analysis in freshwater fish feeding ecology. University of Prešov.

[ece311111-bib-0040] Maret, T. R. , Cain, D. J. , MacCoy, D. E. , & Short, T. M. (2003). Response of benthic invertebrate assemblages to metal exposure and bioaccumulation associated with hard‐rock mining in northwestern streams, USA. Journal of the North American Benthological Society, 22, 598–620.

[ece311111-bib-0041] Marklund, M. H. K. , Svanbäck, R. , Faulks, L. , Breed, M. F. , Scharnweber, K. , Zha, Y. H. , & Eklöv, P. (2019). Asymmetrical habitat coupling of an aquatic predator‐the importance of individual specialization. Ecology and Evolution, 9, 3405–3415. 30962901 10.1002/ece3.4973 PMC6434573

[ece311111-bib-0042] McKnight, D. M. , & Feder, G. L. (1984). The ecological effect of acid conditions and prepipitation of hydrous metal‐oxides in a rocky mountain stream. Hydrobiologia, 119, 129–138.

[ece311111-bib-0043] McKnight, D. T. , Huerlimann, R. , Bower, D. S. , Schwarzkopf, L. , Alford, R. A. , & Zenger, K. R. (2019). Micro Decon: A highly accurate read‐subtraction tool for the post‐sequencing removal of contamination in metabarcoding studies. Environmental DNA, 1, 14–25.

[ece311111-bib-0044] Mehner, T. , Ihlau, J. , Dörner, H. , & Hölker, F. (2005). Can feeding of fish on terrestrial insects subsidize the nutrient pool of lakes? Limnology and Oceanography, 50, 2022–2031.

[ece311111-bib-0045] Nakano, S. , Miyasaka, H. , & Kuhara, N. (1999). Terrestrial‐aquatic linkages: Riparian arthropod inputs alter trophic cascades in a stream food web. Ecology, 80, 2435–2441.

[ece311111-bib-0046] Nakano, S. , & Murakami, M. (2001). Reciprocal subsidies: Dynamic interdependence between terrestrial and aquatic food webs. Proceedings of the National Academy of Sciences of the United States of America, 98, 166–170. 11136253 10.1073/pnas.98.1.166 PMC14562

[ece311111-bib-0047] Olden, J. D. , & Naiman, R. J. (2010). Incorporating thermal regimes into environmental flows assessments: Modifying dam operations to restore freshwater ecosystem integrity. Freshwater Biology, 55, 86–107.

[ece311111-bib-0048] Perkin, E. K. , Hölker, F. , Richardson, J. S. , Sadler, J. P. , Wolter, C. , & Tockner, K. (2011). The influence of artificial light on stream and riparian ecosystems: Questions, challenges, and perspectives. Ecosphere, 2, art122.

[ece311111-bib-0049] Persson, L. (1983). Effects of intra‐ and interspecific competition on dynamics and size structure of a perch *Perca fluviatilis* and a roach *Rutilus rutilus* population. Oikos, 41, 126–132.

[ece311111-bib-0050] Polis, G. A. , & Hurd, S. D. (1996). Linking marine and terrestrial food webs: Allochthonous input from the ocean supports high secondary productivity on small islands and coastal land communities. American Naturalist, 147, 396–423.

[ece311111-bib-0051] R Core Team . (2022). R: A language and environment for statistical computing. R foundation for statistical Computing.

[ece311111-bib-0052] Raat, A. (1988). Synopsis of the biological data on the northern pike *Esox lucius* Linnaeus, 1758. FAO Fisheries Synopsis, 30, 178.

[ece311111-bib-0053] Rasmussen, K. , & Lindegaard, C. (1988). Effects of iron compounds on macroinvertebrate communities in a Danish lowland river system. Water Research, 22, 1101–1108.

[ece311111-bib-0054] Sabater, S. , Elosegi, A. , & Ludwig, R. (2019). Multiple stressors in river ecosystems. Status, impacts and prospects for the future. Elsevier.

[ece311111-bib-0055] Saunders, W. C. , & Fausch, K. D. (2012). Grazing management influences the subsidy of terrestrial prey to trout in central Rocky Mountain streams (USA). Freshwater Biology, 57, 1512–1529.

[ece311111-bib-0056] Scharnweber, K. , Chaguaceda, F. , & Eklöv, P. (2021). Fatty acid accumulation in feeding types of a natural freshwater fish population. Oecologia, 196, 53–63. 33900451 10.1007/s00442-021-04913-y PMC8139920

[ece311111-bib-0057] Scharnweber, K. , & Gårdmark, A. (2020). Feeding specialists on fatty acid‐rich prey have higher gonad weights: Pay‐off in Baltic perch? Ecosphere, 11, 12.

[ece311111-bib-0058] Scharnweber, K. , Syväranta, J. , Hilt, S. , Brauns, M. , Vanni, M. J. , Brothers, S. , Köhler, J. , Knežević‐Jarić, J. , & Mehner, T. (2014). Whole‐lake experiments reveal the fate of terrestrial particulate organic carbon in benthic food webs of shallow lakes. Ecology, 95, 1496–1505. 25039215 10.1890/13-0390.1

[ece311111-bib-0059] Schmutz, S. , & Moog, O. (2018). Dams: Ecological impacts and management. In S. Schmutz & J. Sendzimir (Eds.), Riverine ecosystem management. Springer.

[ece311111-bib-0060] Scholz, C. , & Voigt, C. C. (2022). Diet analysis of bats killed at wind turbines suggests large‐scale losses of trophic interactions. Conservation Science and Practice, 4, e12744.

[ece311111-bib-0061] Soininen, J. , Bartels, P. , Heino, J. , Luoto, M. , & Hillebrand, H. (2015). Toward more integrated ecosystem research in aquatic and terrestrial environments. Bioscience, 65, 174–182.

[ece311111-bib-0062] Solomon, C. T. , Cole, J. J. , Doucett, R. R. , Pace, M. L. , Preston, N. D. , Smith, L. E. , & Weidel, B. C. (2009). The influence of environmental water on the hydrogen stable isotope ratio in aquatic consumers. Oecologia, 161, 313–324. 19471971 10.1007/s00442-009-1370-5

[ece311111-bib-0063] Soto, D. X. , Decru, E. , Snoeks, J. , Verheyen, E. , Van de Walle, L. , Bamps, J. , Mambo, T. , & Bouillon, S. (2019). Terrestrial contributions to Afrotropical aquatic food webs: The Congo River case. Ecology and Evolution, 9, 10746–10757. 31624578 10.1002/ece3.5594 PMC6787788

[ece311111-bib-0064] Soto, D. X. , Koehler, G. , Wassenaar, L. I. , & Hobson, K. A. (2017). Re‐evaluation of the hydrogen stable isotopic composition of keratin calibration standards for wildlife and forensic science applications. Rapid Communications in Mass Spectrometry, 31, 1193–1203. 28475227 10.1002/rcm.7893

[ece311111-bib-0065] Soukup, P. R. , Näslund, J. , Höjesjö, J. , & Boukal, D. S. (2022). From individuals to communities: Habitat complexity affects all levels of organization in aquatic environments. Wiley Interdisciplinary Reviews Water, 9, e1575.

[ece311111-bib-0066] Svanbäck, R. , & Bolnick, D. I. (2007). Intraspecific competition drives increased resource use diversity within a natural population. Proceedings of the Royal Society B: Biological Sciences, 274, 839–844. 10.1098/rspb.2006.0198 PMC2093969 17251094

[ece311111-bib-0067] Tanentzap, A. J. , Szkokan‐Emilson, E. J. , Kielstra, B. W. , Arts, M. T. , Yan, N. D. , & Gunn, J. M. (2014). Forests fuel fish growth in freshwater deltas. Nature Communications, 5, 9. 10.1038/ncomms5077 PMC4082636 24915965

[ece311111-bib-0068] Twining, C. W. , Bernhardt, J. R. , Derry, A. M. , Hudson, C. M. , Ishikawa, A. , Kabeya, N. , Kainz, M. J. , Kitano, J. , Kowarik, C. , Ladd, S. N. , Leal, M. C. , Scharnweber, K. , Shipley, J. R. , & Matthews, B. (2021). The evolutionary ecology of fatty‐acid variation: Implications for consumer adaptation and diversification. Ecology Letters, 24, 1709–1731. 34114320 10.1111/ele.13771

[ece311111-bib-0069] Twining, C. W. , Brenna, J. T. , Hairston, N. G. , & Flecker, A. S. (2016). Highly unsaturated fatty acids in nature: What we know and what we need to learn. Oikos, 125, 749–760.

[ece311111-bib-0070] Uhlig, U. , Radigk, S. , Uhlmann, W. , Preuß, V. , & Koch, T. (2016). Iron removal from the Spree River in the Bühlow pre‐impoundment basin of the Spremberg reservoir. In C. Drebenstedt & M. Paul (Eds.), IMWA 2026‐Mining meets water‐conflicts and solutions (pp. 182–190). Technische Universität Bergakademie Freiberg.

[ece311111-bib-0071] Uhlmann, W. R. , Hiekel, R. , & Giering, N. (2021). *Monitoring der Eisenbelastung in der Spree und in der Talsperre Spremberg, Jahresbericht 2020*. Institut für Wasser und Boden Dr, Uhlmann, im Auftrag der Lausitzer und Mitteldeutschen Bergbau‐Verwaltungsgesellschaft mbH, Dresden.

[ece311111-bib-0072] Vander Zanden, H. B. , Soto, D. X. , Bowen, G. J. , & Hobson, K. A. (2016). Expanding the isotopic toolbox: Applications of hydrogen and oxygen stable isotope ratios to food web studies. Frontiers in Ecology and Evolution, 4, 20.

[ece311111-bib-0073] Vuori, K. M. (1995). Direct and indirect effects of iron on river ecosystems. Annales Zoologici Fennici, 32, 317–329.

[ece311111-bib-0074] Wassenaar, L. I. , & Hobson, K. A. (2003). Comparative equilibration and online technique for determination of non‐exchangeable hydrogen of keratins for use in animal migration studies. Isotopes in Environmental and Health Studies, 39, 211–217. 14521282 10.1080/1025601031000096781

[ece311111-bib-0075] Závorka, L. , Blanco, A. , Chaguaceda, F. , Cucherousset, J. , Killen, S. S. , Liénart, C. , Mathieu‐Resuge, M. , Němec, P. , Pilecky, M. , Scharnweber, K. , Twining, C. W. , & Kainz, M. J. (2022). The role of vital dietary biomolecules in eco‐evo‐devo dynamics. Trends in Ecology & Evolution, 38, 72–84. 36182405 10.1016/j.tree.2022.08.010

