# Supplementary material for: Effects of mining activities on fish communities and food web dynamics in a lowland river
Source: Ecol Evol. 2024 Mar 11;14(3):e11111. doi: 10.1002/ece3.11111 (PMC10928357; doi:10.1002/ece3.11111)
Supplement: Supplementary file 1 — Appendix S1 [file ECE3-14-e11111-s001.docx]

**Supplementary Material**

**Appendix 1**

Abundance of benthic macroinvertebrate taxa (individuals m^-2^) collected at the three locations upstream (i.e., high iron concentrations) and the three locations downstream (i.e., low iron concentrations) of the dam.

|  | **Upstream of the dam** | | | **Downstream of the dam** | | |
| --- | --- | --- | --- | --- | --- | --- |
| **site** | **Trattendorf** | **Wilhelmstal** | **Zerre** | **Bräsinchen** | **Frauendorf** | **Madlow** |
| Odonata | 0 | 0 | 0 | 59 | 74 | 0 |
| Trichoptera | 89 | 0 | 44 | 444 | 222 | 44 |
| Chironomidae | 178 | 2102 | 118 | 281 | 755 | 2590 |
| other Diptera | 59 | 15 | 15 | 30 | 340 | 178 |
| Bivalvia | 44 | 30 | 44 | 3286 | 696 | 178 |
| Gammaridae | 0 | 30 | 15 | 133 | 118 | 0 |
| Oligochaeta | 15 | 15 | 44 | 44 | 15 | 104 |
| Gastropoda | 0 | 15 | 0 | 2190 | 5343 | 0 |
| Ephemeroptera | 0 | 0 | 0 | 0 | 59 | 533 |
| Heteroptera | 0 | 0 | 0 | 0 | 15 | 0 |
| Plecoptera | 15 | 0 | 0 | 0 | 59 | 0 |
| Megaloptera | 15 | 44 | 0 | 0 | 44 | 0 |
| **Total** | 414 | 2250 | 281 | 6468 | 7740 | 3626 |

**Appendix 2**

a) Abundance (represented as catch per unit effort, CPUE, individuals 1000 m^-1^) and b) biomass of fish species (represented as biomass per unit effort, BPUE, g 1000 m^-1^) collected at the three locations upstream (i.e., high iron concentrations) and the three locations downstream (i.e., low iron concentrations) of the dam.

a) CPUE (individuals 1000 m^-1^)

|  | **Upstream of the dam** | | | **Downstream of the dam** | | |
| --- | --- | --- | --- | --- | --- | --- |
| **site** | **Trattendorf** | **Wilhelmstal** | **Zerre** | **Bräsinchen** | **Frauendorf** | **Madlow** |
| Barbel *Barbus barbus* | 0 | 0 | 0 | 0 | 0 | 0.7 |
| Bitterling *Rhodeus amarus* | 1.9 | 0 | 0 | 2.2 | 8.3 | 0 |
| Bleak *Alburnus alburnus* | 4.9 | 16.9 | 2.8 | 26.2 | 35.5 | 28.3 |
| Catfish *Silurus glanis* | 0.0 | 0.3 | 0.6 | 0.3 | 0.0 | 0 |
| Chub *Squalius cephalus* | 7.7 | 2.1 | 1.7 | 1.2 | 6.2 | 6.3 |
| Crucian carp *Carassius carassius* | 0 | 0 | 0 | 0.3 | 0 | 0 |
| Common dace *Leuciscus leuciscus* | 0 | 0.5 | 0 | 0.6 | 0 | 0.3 |
| European eel *Anguilla anguilla* | 0 | 2.3 | 1.1 | 9.9 | 1.5 | 1.7 |
| Gudgeon *Gobio gobio* | 5.2 | 17.2 | 1.1 | 0 | 0.3 | 1.7 |
| River lamprey *Lampetra fluviatilis* | 0 | 0 | 0.6 | 0 | 0 | 0 |
| European perch *Perca fluviatilis* | 4.3 | 7.0 | 2.2 | 68.2 | 19.1 | 1.7 |
| Pike *Esox lucius* | 2.8 | 3.9 | 3.9 | 4.6 | 0.6 | 2.3 |
| Roach *Rutilus rutilus* | 17.6 | 40.4 | 15.0 | 41.4 | 41.7 | 90.3 |
| Rudd *Scardinius erythrophtalmus* | 2.2 | 1.0 | 1.1 | 10.5 | 0.6 | 0 |
| Ruffe *Gymnocephalus cernua* | 0 | 0 | 0 | 0.9 | 0 | 0.3 |
| Stone loach *Barbatula barbatula* | 0.3 | 0 | 0 | 0 | 0 | 0 |
| Tench *Tinca tinca* | 0 | 0 | 0 | 3.4 | 0.6 | 0.3 |
| Three-spined stickleback  *Gasterosteus aculeatus* | 2.2 | 0 | 1.1 | 4.6 | 0 | 0 |
| White bream *Blicca bjoerkna* | 0 | 0.8 | 0.6 | 38.9 | 6.8 | 6.7 |
| Zander *Sander lucioperca* | 0 | 0.3 | 0 | 0 | 0 | 0 |
| Juveniles, not specified | 0 | 6.3 | 0 | 0 | 0 | 0 |
| **Total** | **49.1** | **99.0** | **31.7** | **213.3** | **121.3** | **140.7** |

b) BPUE (g 1000 m^-1^)

|  | **Upstream of the dam** | | | **Downstream of the dam** | | |
| --- | --- | --- | --- | --- | --- | --- |
| **site** | **Trattendorf** | **Wilhelmstal** | **Zerre** | **Bräsinchen** | **Frauendorf** | **Madlow** |
| Barbel *Barbus barbus* | 0 | 0 | 0 | 0 | 0 | 194 |
| Bitterling *Rhodeus amarus* | 0 | 0 | 0 | 1.8 | 14.5 | 0 |
| Bleak *Alburnus alburnus* | 3.6 | 78.2 | 57.6 | 59.4 | 14.2 | 264.2 |
| Catfish *Silurus glanis* | 0 | 45.7 | 99.7 | 5.4 | 0 | 0 |
| Chub *Squalius cephalus* | 21.7 | 4.6 | 44.7 | 285.7 | 73.7 | 1664.1 |
| Crucian carp *Carassius carassius* | 0 | 0 | 0 | 3.5 | 0 | 0 |
| Common dace *Leuciscus leuciscus* | 0 | 4.3 | 0 | 48.5 | 0 | 27.3 |
| European eel *Anguilla anguilla* | 0 | 63.3 | 261.6 | 1209.1 | 162 | 91.6 |
| Gudgeon *Gobio gobio* | 0 | 53.6 | 5.6 | 0 | 0 | 33.4 |
| River lamprey *Lampetra fluviatilis* | 0 | 0 | 2.2 | 0 | 0 | 0 |
| European perch *Perca fluviatilis* | 83.6 | 131.9 | 25.1 | 512.8 | 397.1 | 44.8 |
| Pike *Esox lucius* | 372.6 | 533.4 | 1451.9 | 494.5 | 30 | 2564.2 |
| Roach *Rutilus rutilus* | 170.5 | 376.8 | 181.9 | 1209.4 | 798.6 | 4293.8 |
| Rudd *Scardinius erythrophtalmus* | 32.3 | 7.4 | 35.1 | 40.5 | 10.6 | 0 |
| Ruffe *Gymnocephalus cernua* | 0 | 0 | 0 | 2.1 | 0 | 0.8 |
| Stone loach *Barbatula barbatula* | 0.8 | 0 | 0 | 0 | 0 | 0 |
| Tench *Tinca tinca* | 0 | 0 | 0 | 274.4 | 203.7 | 36.5 |
| Three-spined stickleback  *Gasterosteus aculeatus* | 0 | 0 | 1.1 | 0.4 | 0 | 0 |
| White bream *Blicca bjoerkna* | 0 | 29.6 | 43.9 | 49.5 | 10.1 | 477.6 |
| Zander *Sander lucioperca* | 0 | 2.8 | 0 | 0 | 0 | 0 |
| Juveniles, not specified | 0 | 0.6 | 0 | 0 | 0 | 0 |
| **Total** | 685.1 | 1332.3 | 2210.2 | 4196.8 | 1714.4 | 9692 |

**Appendix 3**

Number of fish individuals upstream and downstream of the dam with the different a) aquatic, and b) terrestrial species in the gut content, identified by DNA metabarcoding.

|  |  | **Upstream of the dam**  **(high iron concentrations)** | | | **Downstream of the dam**  **(low iron concentrations)** | | |
| --- | --- | --- | --- | --- | --- | --- | --- |
|  |  | perch  (*N* = 4) | bleak  (*N* = 4) | roach  (*N* = 4) | perch  (*N* =4) | bleak  (*N* = 6) | roach  (*N* = 9) |
| 1. **Aquatic species** |  |  |  |  |  |  |  |
| Acari | *Eupelops sp.* | 1 | 0 | 0 | 0 | 0 | 0 |
| Actinopteri | *Esox lucius* | 0 | 0 | 0 | 1 | 0 | 0 |
| Amphipoda | *Gammarus fossarum* | 0 | 1 | 0 | 0 | 0 | 0 |
| Cyclopoida | *Mesocyclops leuckarti* | 0 | 0 | 0 | 1 | 0 | 0 |
|  | *Thermocyclops oithonoides* | 0 | 0 | 0 | 1 | 0 | 5 |
| Coleoptera | *Deronectes latus* | 1 | 0 | 0 | 0 | 0 | 0 |
| Cyclopoida | *Eucyclops serrulatus* | 0 | 2 | 0 | 0 | 0 | 0 |
|  | *Paracyclops fimbriatus* | 0 | 1 | 0 | 0 | 0 | 0 |
| Diplostraca | *Daphnia cucullata* | 0 | 0 | 0 | 1 | 2 | 6 |
|  | *Daphnia galeata* | 1 | 0 | 0 | 3 | 4 | 7 |
|  | *Diaphanosoma brachyurum* | 0 | 0 | 0 | 3 | 4 | 7 |
| Diptera | *Anopheles claviger* | 1 | 0 | 0 | 0 | 0 | 0 |
|  | *Chironomus plumosus* | 0 | 0 | 0 | 0 | 0 | 1 |
|  | *Chironomus riparius* | 0 | 2 | 0 | 0 | 0 | 0 |
|  | *Culiseta morsitans* | 1 | 0 | 0 | 0 | 0 | 0 |
|  | *Einfeldia dissidens* | 1 | 0 | 2 | 0 | 1 | 1 |
|  | *Microchironomus tener* | 0 | 0 | 0 | 0 | 1 | 0 |
|  | *Phaenopsectra sp.* | 1 | 0 | 0 | 0 | 0 | 0 |
|  | *Culiseta morsitans* | 1 | 0 | 0 | 0 | 0 | 0 |
|  | *Tanytarsus eminulus* | 0 | 0 | 0 | 0 | 0 | 2 |
| Gastropoda | *Physella acuta* | 0 | 0 | 0 | 1 | 0 | 2 |
|  | *Potamopyrgus antipodarum* | 0 | 0 | 0 | 0 | 0 | 1 |
|  | *Radix balthica* | 0 | 0 | 1 | 0 | 0 | 0 |
| Haplotaxida | *Aulodrilus pluriseta* | 1 | 0 | 0 | 0 | 0 | 0 |
| Hemiptera | *Micronecta griseola* | 0 | 0 | 1 | 0 | 0 | 0 |
| Hydrozoa | *Hydra oligactis* | 0 | 0 | 0 | 0 | 0 | 1 |
| Isopoda | *Asellus aquaticus* | 1 | 0 | 0 | 0 | 0 | 0 |
| Ploima | *Keratella quadrata* | 0 | 0 | 0 | 0 | 0 | 1 |
| Trichoptera | *Hydropsyche pellucidula* | 0 | 0 | 0 | 0 | 1 | 1 |
|  | *Neureclipsis bimaculata* | 0 | 0 | 0 | 2 | 1 | 0 |
|  | *Oecetis notata* | 0 | 0 | 0 | 0 | 0 | 1 |
| Unionicolidae | *Unionicola minor* | 0 | 0 | 0 | 0 | 0 | 1 |
|  |  |  |  |  |  |  |  |
| 1. **Terrestrial species** |  |  |  |  |  |  |  |
| Aranae | *Philodromus aureolus* | 1 | 0 | 0 | 0 | 0 | 0 |
| Coleoptera | *Curculio glandium* | 1 | 0 | 0 | 0 | 0 | 0 |
| Dermaptera | *Forficula auricularia* | 1 | 0 | 0 | 0 | 0 | 0 |
| Diptera | *Medetera sp.* | 2 | 0 | 0 | 0 | 0 | 0 |
|  | *Sarcophaga variegata* | 1 | 0 | 0 | 0 | 0 | 0 |
|  | *Siphona pauciseta* | 1 | 0 | 0 | 0 | 0 | 0 |
|  | *Thricops simplex* | 1 | 0 | 0 | 0 | 0 | 0 |
|  | *Tolmerus atricapillus* | 1 | 0 | 0 | 0 | 0 | 0 |
| Entomobryidae | *Lepidocyrtus paradoxus* | 1 | 0 | 0 | 0 | 0 | 0 |
| Hemiptera | *Elasmucha grisea* | 0 | 1 | 0 | 0 | 0 | 0 |
| Hymenoptera | *Formica aquilonia* | 0 | 1 | 0 | 0 | 0 | 0 |
|  | *Lasius niger* | 0 | 1 | 0 | 0 | 0 | 0 |
| Isopoda | *Armadillidium vulgare* | 2 | 0 | 0 | 0 | 0 | 0 |
|  | *Porcellio scaber* | 1 | 0 | 0 | 0 | 0 | 0 |
| Lepidoptera | *Dypterygia scabriuscula* | 1 | 0 | 0 | 0 | 0 | 0 |
| Lumbricidae | *Aporrectodea caliginosa* | 1 | 0 | 0 | 0 | 0 | 0 |
|  | *Dendrobaena octaedra* | 1 | 0 | 0 | 0 | 0 | 0 |

**Appendix 4**

Averages ± standard deviations of δ^2^H (‰) of samples used in this study. Corrected values represent the environmental water corrected stable isotope ratio using the equation (2) of (Brett et al. 2018) with ω= 0.28 (Soto et al. 2019) and -55.06 ‰ for δ^2^H of the river water (Chen et al. 2023).

|  | **Seston**  **(‰)** | **terrestrial insects (‰)** | **perch**  **(raw/ corrected)**  **(‰)** | **bleak**  **(raw/ corrected)**  **(‰)** | **roach**  **(raw/ corrected)**  **(‰)** |
| --- | --- | --- | --- | --- | --- |
| Upstream of the dam | -184.7 ± 0.7 | -94.8 ± 16.7  -79.7 ± 16.7 | -94.1 ± 7.5  -73.1 ± 7.5 | -96.6 ± 7.1  -93.3 ± 3.5 | -105.1 ± 10.6  -75.6 ± 7.1 |
| Downstream of the dam | -201.7 ± 4.2 | -92.9 ± 9.6  -77.8 ± 9.6 | -125.8 ± 4.8  -104.8 ± 4.8 | -127.7 ± 5.6  -106.7 ± 5.6 | -121.7 ± 3.7  -100.7 ± 3.7 |

**Appendix 5**

Water temperatures at the different sites upstream (Trattendorf, Wilhelmstal, Zerre) and downstream of the dam (Bräsinchen, Frauendorf, Madlow) between April 2020 and August 2021. Temperature loggers (HOBO, OneTemp Pty Ltd., Adelaide, Australia) were placed above the substrate and water temperature was recorded every 6 hours.


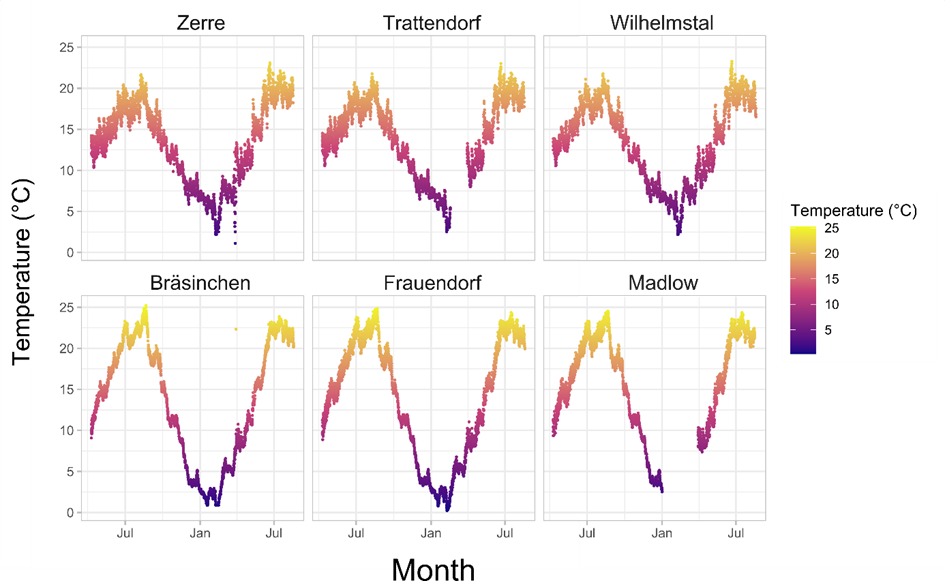


**Reference list**

Brett, M. T., G. W. Holtgrieve, and D. E. Schindler. 2018. An assessment of assumptions and uncertainty in deuterium-based estimates of terrestrial subsidies to aquatic consumers. Ecology **99**:1073-1088.

Chen, K., D. Tetzlaff, T. Goldhammer, J. Freymueller, S. Wu, S. A.A., A. Schmidt, G. Liu, M. Venohr, and C. Soulsby. 2023. Synoptic water isotope surveys to understand the hydrology of large intensively managed catchments. Journal of Hydrology **623**.

Soto, D. X., E. Decru, J. Snoeks, E. Verheyen, L. Van de Walle, J. Bamps, T. Mambo, and S. Bouillon. 2019. Terrestrial contributions to Afrotropical aquatic food webs: The Congo River case. Ecology and Evolution **9**:10746-10757.
